# S-phase Synchronization Facilitates the Early Progression of Induced-Cardiomyocyte Reprogramming through Enhanced Cell-Cycle Exit

**DOI:** 10.3390/ijms19051364

**Published:** 2018-05-04

**Authors:** Emre Bektik, Adrienne Dennis, Gary Pawlowski, Chen Zhou, Danielle Maleski, Satoru Takahashi, Kenneth R. Laurita, Isabelle Deschênes, Ji-Dong Fu

**Affiliations:** 1 Department of Medicine, Heart and Vascular Research Center, MetroHealth Campus, Case Western Reserve University, Cleveland, OH 44109, USA; exb329@case.edu (E.B.); adennis@metrohealth.org (A.D.); gpawlowski@metrohealth.org (G.P.); cxz399@case.edu (C.Z.); dmaleski@metrohealth.org (D.M.); kenneth.laurita@case.edu (K.R.L.); isabelle.deschenes@case.edu (I.D.); 2 Ph.D. Program in Human Biology, School of Integrative and Global Majors, University of Tsukuba, Tsukuba 305-8577, Japan; satoruta@md.tsukuba.ac.jp; 3 Department of Anatomy and Embryology, Faculty of Medicine, University of Tsukuba, Tsukuba 305-8577, Japan

**Keywords:** induced cardiomyocyte, epigenetic reprogramming, cell division, cell-cycle synchronization, cell-cycle exit

## Abstract

Direct reprogramming of fibroblasts into induced cardiomyocytes (iCMs) holds a great promise for regenerative medicine and has been studied in several major directions. However, cell-cycle regulation, a fundamental biological process, has not been investigated during iCM-reprogramming. Here, our time-lapse imaging on iCMs, reprogrammed by Gata4, Mef2c, and Tbx5 (GMT) monocistronic retroviruses, revealed that iCM-reprogramming was majorly initiated at late-G1- or S-phase and nearly half of GMT-reprogrammed iCMs divided soon after reprogramming. iCMs exited cell cycle along the process of reprogramming with decreased percentage of 5-ethynyl-20-deoxyuridine (EdU)^+^/α-myosin heavy chain (αMHC)-GFP^+^ cells. S-phase synchronization post-GMT-infection could enhance cell-cycle exit of reprogrammed iCMs and yield more GFP^high^ iCMs, which achieved an advanced reprogramming with more expression of cardiac genes than GFP^low^ cells. However, S-phase synchronization did not enhance the reprogramming with a polycistronic-viral vector, in which cell-cycle exit had been accelerated. In conclusion, post-infection synchronization of S-phase facilitated the early progression of GMT-reprogramming through a mechanism of enhanced cell-cycle exit.

## 1. Introduction

Cardiomyocytes (CMs) in the adult heart have limited regenerative capacity [1]. At the onset of heart disease, lost CMs are typically replaced with fibrotic scar tissue, subsequently leading to chronic heart failure, which remains one of the leading causes of death worldwide. Recent studies have found that mouse [2,3,4,5] and human [6,7,8,9] fibroblasts can be directly reprogrammed into induced CMs (iCMs), which holds a great promise to develop a new therapeutic approach for heart disease. In order to improve induction efficiency and quality of iCMs, studies have focused on developing optimized reprogramming methods and investigating the mechanism of direct cardiac reprogramming, including optimized gene-delivery approaches of reprogramming factors [10,11], suppression of critical epigenetic barriers [12,13] and pro-fibrotic signaling [14,15,16], and optimization of culture conditions [17,18]. However, the cell-cycle regulation, a fundamental biological process, has not been investigated during iCM-reprogramming.

Cell cycle regulation and cell-cycle exit constitute important events of iCM-reprogramming. Similar to fully differentiated adult CMs, it has been recognized that reprogrammed iCMs exit the cell cycle. No cardiac troponin-T (cTnT)^+^ iCMs were positively stained with Ki67 at week-2 of reprogramming [19]; 5-ethynyl-20-deoxyuridine (EdU) assay did not show any EdU^+^ iCMs from week-2 to week-4 post-induction [17]. More recently, none of the α-Actinin^+^ iCMs expressed proliferation marker, Ki67 four weeks after reprogramming [20]. However, it is unknown whether cell-cycle exit of reprogrammed iCMs happens right upon reprogramming induction or at a later stage of reprogramming process. A cell cycle constitutes a critically important chain of interconnected events with a dynamic fluctuation of epigenetic chromatin modifications [21], including genomic DNA methylation and histone modification, which have significant influence on the epigenetic reprogramming of somatic cell fate [22]. Indeed, it has been reported that pre-synchronization of fibroblasts at the G0/G1-phase by transient serum starvation could significantly improve the reprogramming yield of induced pluripotent stem cells (iPSCs) [23]. In addition, cell-cycle pre-synchronization at the G1-phase could markedly enhance the reprogramming efficiency of induced dopaminergic neurons [24]. These studies suggested that manipulation of cell-cycle progression has a significant impact on epigenetic reprogramming, however, it is unknown whether a particular phase of cell cycle favors the initiation of iCM- reprogramming and if manipulating the cell cycle (i.e., synchronization) of post-infected fibroblasts influences the progression of reprogramming.

In this study, transgenic mouse embryonic fibroblasts (MEFs), in which the expression of GFP is controlled by the promoter of α-myosin heavy chain (αMHC-GFP), were used for iCM-reprogramming. We first performed 48-h time-lapse recordings to monitor the early progression of iCM-reprogramming and found that αMHC-GFP^+^ iCMs went through cell division at the early stage of reprogramming. We calculated the time from the initial expression of αMHC-GFP to cell division and estimated at which cell-cycle phase iCM-reprogramming was initiated. After we confirmed that iCMs exited cell cycle along the process of reprogramming, we synchronized cell cycle of fibroblasts at various time points of post-retrovirus infection and found that a post-infection synchronization of S-phase enhanced cell-cycle exit of reprogrammed iCMs and accelerated the early progression of reprogramming.

## 2. Results

### 2.1. iCMs Go through Cell Division and Exit Cell Cycle Along with the Progress of Reprogramming

**Figure 1:**
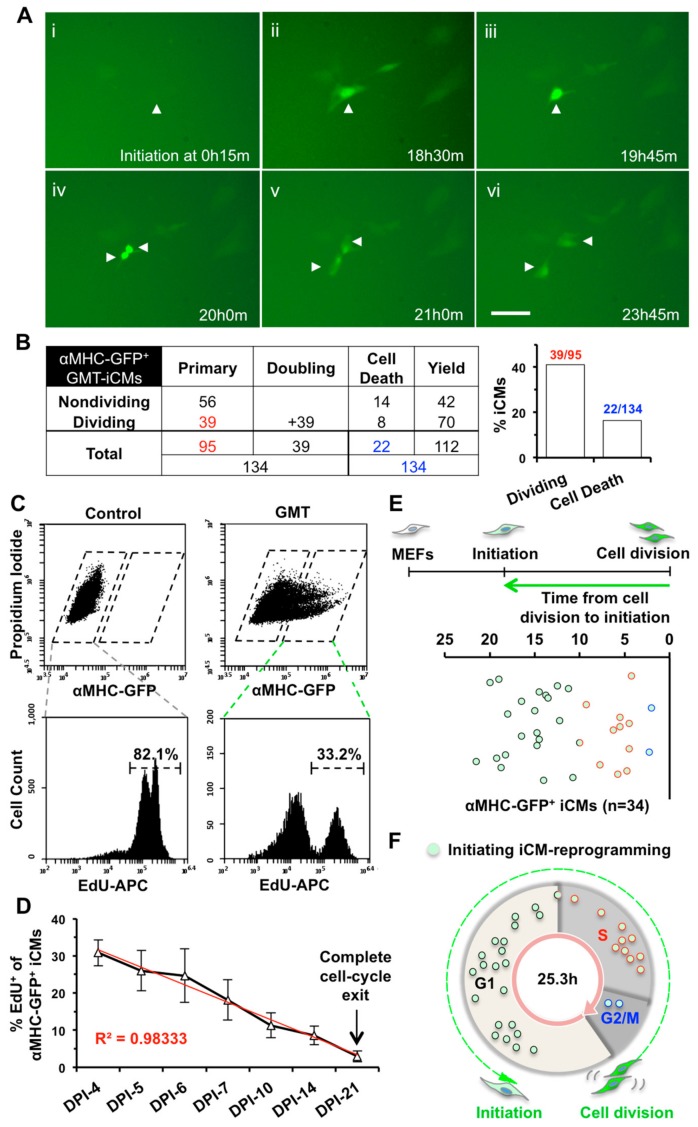
Induced Cardiomyocytes (iCMs) undergo cell division and exit cell cycle along the process of reprogramming. (**A**) Representative images of a time-lapse recording of GMT-reprogramming showing that one primary iCM (arrowhead) divided into two daughter iCMs 20.75 h after the activation of αMHC-GFP. A scale bar indicates 50 µm. (**B**) A table summarizing all three batches time-lapse results of GMT-iCMs. We used these numbers in red to calculate the percentage of dividing primary iCMs and numbers in blue for the percentage of cell death among total iCMs, which were summarized in the bar graph. (**C**) Representative FACS plots of αMHC-GFP^+^ iCMs (upper panels) and of 24-h-incubation EdU assay assessing cell division of Mouse Embryonic Fibroblasts (MEFs) (lower-left panel) and αMHC-GFP^+^ iCMs (lower-right panel) at day-4 post-infection (DPI-4). (**D**) Percentage of dividing EdU^+^/αMHC-GFP^+^ GMT-iCMs from DPI-4 to DPI-21 (*n* = 5). (**E**) The time duration from the reprogramming-initiation to cell division in dividing GMT-iCMs (*n* = 34 from three batches). (**F**) A cell-cycle-distribution chart of dividing iCMs (panel E) at the time point of reprogramming initiation.

For iCM reprogramming, we infected transgenic αMHC-GFP MEFs with a cocktail of monocistronic Gata4, Mef2c, and Tbx5 (GMT) retroviruses and found that GFP could be first observed from day 2 post-infection (DPI-2), which was consistent with the observation that a high-level overexpression of GMT was achieved around 48 h post-infection (Figure S1A). We recorded a 48-h time-lapse at DPI-2 through DPI-4 to monitor the activation of αMHC-GFP during the early progression of iCM-reprogramming and to determine if cell division occurs during iCM-reprogramming. We purposely set a three-second-exposure time for GFP recording to recognize very faint αMHC-GFP fluorescence, indicative of initial activation of reprogramming (Figure 1A, frame İ). We found that the fluorescence of αMHC-GFP was gradually enhanced during the process of reprogramming. Surprisingly, we found that ~41% (39 out of 95) of αMHC-GFP^+^ primary GMT-reprogrammed iCMs (GMT-iCMs) underwent cell division within the 48-h recording time (Figure 1A,B, Movie S1). Noticeably, ~16% (22 out of 134) of GMT-iCMs died before or after cell division (Figure 1B). Our time-lapse recordings revealed that iCMs at the early stage of reprogramming could still actively divide.

We next performed an EdU assay to quantify cell division of αMHC-GFP^+^ iCMs from DPI-4 to later stages of the reprogramming process. Consistent with that in cardiac fibroblasts [2], the percentage of αMHC-GFP^+^ iCMs, reprogrammed from MEFs, gradually increased from DPI-4 to DPI-7 and then decreased after two weeks (Figure S1B). We incubated retrovirus-infected MEFs with EdU for 24 h to label all the dividing cells within that time and found that more than 80% of uninfected MEFs had gone through cell division within 24 h (Figure 1C). Noticeably, 30.8 ± 3.5% of GMT-iCMs at DPI-4 entered cell division and was positively stained for EdU, which is consistent with our time-lapse results (DPI-2 to DPI-4). Furthermore, the percentage of EdU^+^-iCMs gradually decreased from DPI-4 to DPI-21 and almost none of the αMHC-GFP^+^ iCMs at DPI-21 were stained positively for EdU (*n* = 5, Figure 1D), indicating that all iCMs, which were αMHC-GFP^+^/EdU^−^, had exited cell cycle at this late stage of reprogramming.

### 2.2. iCM-Reprogramming Is Predominantly Initiated at late-G1- and S-phase

We next asked in which phase of the cell cycle iCM-reprogramming is initiated. To answer this question, we carefully calculated the time between two consecutive cell divisions of MEFs in our time-lapse recordings and estimated that MEFs had an average of 25.3 ± 7.4 h of cell-cycle length (*n* = 42, Figure S1C). We performed EdU assay with two-hour EdU-labeling and measured the average percentages of G1 (~60%), S (~29%), and G2/M (~11%) in MEFs (Figure S1D,E, *n* = 4), which represent the percentages of the time spent in each phase out of whole cell-cycle duration [25]. Therefore, the duration of G1 phase was calculated as ~15.2 h (~60% of 25.3 h), S phase ~7.3 h, and G2/M phase ~2.8 h (Figure S1F). We then measured the time from the completed cell-division back to the first appearance of the αMHC-GFP reporter (Figure 1E, Table S1) and determined in which cell-cycle phase reprogramming of individual iCMs was initiated. For example, the reprogramming initiation of one iCM in Figure 1A (indicated by arrow head) was started from 15 min with the first appearance of faint GFP-fluorescence (Figure 1A, frame i) and cell division happened at 21 h (Figure 1A, frame V). Therefore, reprogramming of this iCM was initiated at the G1 phase and took 20.75 h to pass through G1 (10.65 h), S (7.3 h), and G2/M (2.8 h) phases for a completion of cell division. These transition times from reprogramming initiation to cell division of GMT-iCMs (*n* = 34, Figure 1E) were converted into a distribution chart of cell-cycle phases. We found that 23 iCMs initiated the activation of αMHC-GFP at G1-phase, including 15 at late-G1-phase, 10 at S-phase, and 2 at G2/M-phase (Figure 1F), suggesting that iCM-reprogramming was mostly initiated at late-G1- and S-phase.

### 2.3. S- or G2/M-Phase Synchronization at DPI-1 Facilitates Cell-Cycle Exit of GMT-iCMs

**Figure 2:**
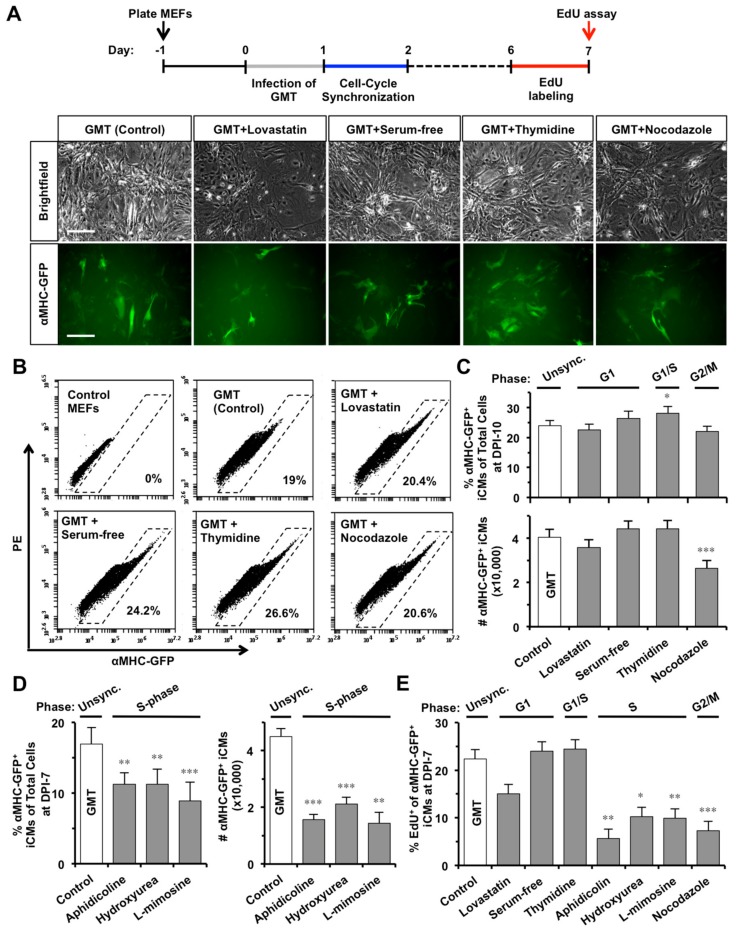
S- or G2/M-phase synchronization at DPI-1 enhances cell-cycle exit in GMT-reprogrammed iCMs. (**A**) At DPI-1, MEFs were synchronized at G1, G0/G1, G1/S, or G2/M-phase by lovastatin, serum-free media, thymidine, or nocodazole, respectively. Representative pictures showing GMT-reprogrammed MEFs at DPI-10 with or without (Control) cell-cycle synchronization. Scale bars indicate 100 µm. (**B**) Representative FACS plots of reprogrammed αMHC-GFP^+^ iCMs at DPI-10. (**C**) The effect of G1-, G1/S-, or G2/M-phase synchronization on GMT-iCMs (*n* = 10), including the percentage (upper panel) and absolute number (lower panel) of αMHC-GFP^+^ iCMs at DPI-10. (**D**) The effect of S-phase synchronization by aphidicolin, hydroxyurea, or L-mimosine on GMT-iCMs (*n* = 5) at DPI-7. (**E**) The percentage of EdU^+^ cells in αMHC-GFP^+^ iCM-population at DPI-7 with or without cell-cycle synchronization at DPI-1 (*n* = 3). * *p* < 0.05, ** *p* < 0.01, *** *p* < 0.001 vs. GMT group.

Since the epigenetic status dynamically fluctuates throughout the cell cycle [21], we then investigated if synchronizing a specific cell-cycle phase in GMT-infected fibroblasts could improve iCM-reprogramming. At DPI-1, GMT-infected MEFs were synchronized at G1-, G0/G1-, G1/S-, or G2/M-phase, by a 24-h incubation with lovastatin, serum-free media, thymidine, or nocodazole (Figure 2A), respectively. The morphology of synchronized MEFs displayed cell-cycle related changes (Figure S2A), as previously reported [26]. We found that thymidine-induced G1/S-synchronization could increase the percent of reprogrammed αMHC-GFP^+^ iCMs, while lovastatin-induced G1 synchronization had no significant influence (Figure 2B,C). However, the absolute number (i.e., yield) of αMHC-GFP^+^ iCMs was not significantly improved by thymidine-synchronization (*n* = 10, Figure 2C) but was dramatically decreased by G2/M-synchronization of nocodazole.

We also investigated the effect of S-phase synchronization (Figure S2A), mediated by aphidicolin, hydroxyurea, or l-mimosine, [27] on iCM-reprogramming and found that all three compounds significantly suppressed iCM-reprogramming with decreased percentage and absolute number of αMHC-GFP^+^ iCMs (*n* = 5, Figure 2D). None of the synchronization treatments inhibited the protein expressions of GMT in infected MEFs (Figure S2B). While un-reprogrammed MEFs could quickly recover from cell-cycle arrest and reenter cell cycle 24 h after removing compounds (Figure S2A), interestingly, we found that S- (aphidicolin, *p* = 0.0039; hydroxyurea, *p* = 0.0163; l-mimosine, *p* = 0.0032) or G2/M-synchronization (nocodazole, *p* = 0.00009), but not G1-synchronization, at DPI-1 significantly decreased the percentage of dividing EdU^+^/αMHC-GFP^+^ GMT-iCMs at DPI-7 (*n* = 3, Figure 2E). Our data suggested that S- or G2/M-synchronization at DPI-1 decreased iCM yield by enhancing cell-cycle exit in GMT-reprogrammed iCMs.

### 2.4. S-phase Synchronization Accelerates the Early Progression of iCM-Reprogramming

**Figure 3:**
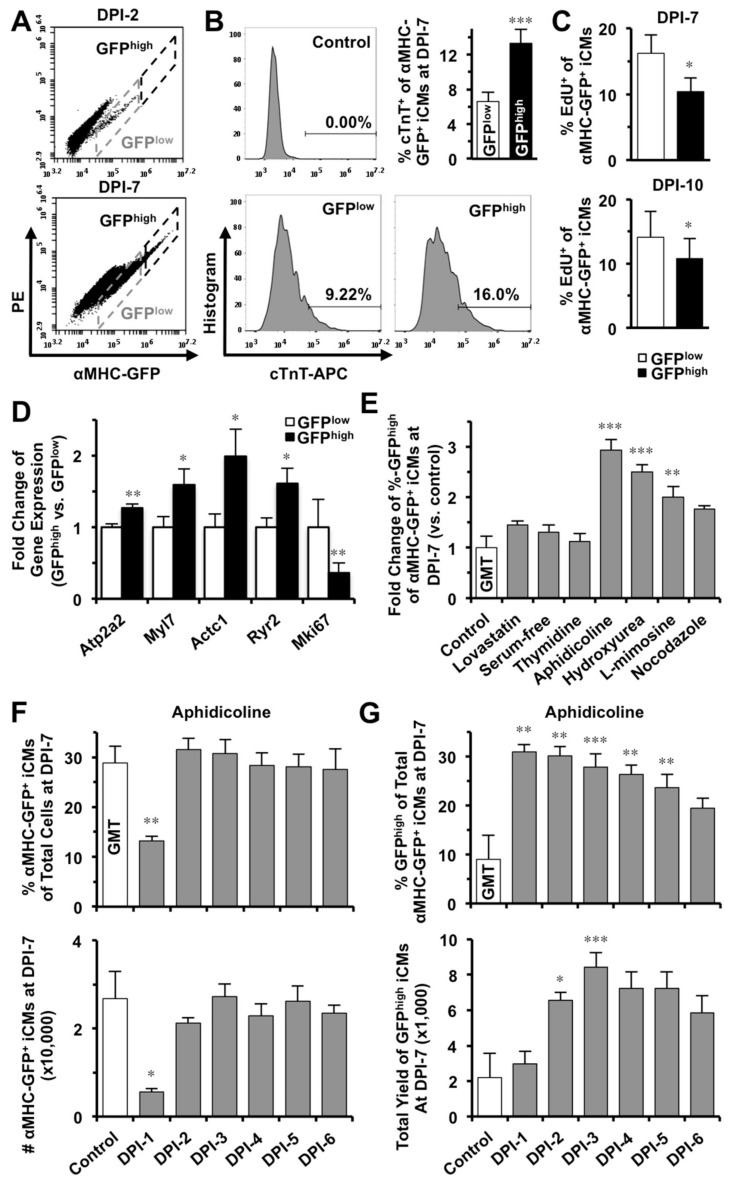
S-phase synchronization accelerates the early progression of reprogramming and increases the yield of GFP^high^ iCMs. (**A**) Reprogrammed iCMs were classified into GFP^low^ and GFP^high^ populations. (**B**) GFP^high^ iCMs contain cardiac troponin T^+^ cells significantly more than GFP^low^ iCMs (*n* = 5, *p* = 0.0005) at DPI-7. (**C**) Significantly less GFP^high^ iCMs were stained positive for EdU than GFP^low^ cells at DPI-7 (*n* = 3) and DPI-10 (*n* = 6). (**D**) Comparisons of gene expression in GFP^low^ and GFP^high^ iCMs at DPI-7 (*n* = 6). (**E**) Only synchronization of S-phase (*n* = 6), but not of other-phases (*n* = 3), at DPI-1 significantly increased GFP^high^ population of GMT-iCMs at DPI-7. (**F**) The effect of S-phase synchronization by aphidicolin (*n* = 3) from DPI-1 to DPI-6 on the percentage and absolute number of GMT-iCMs. (**G**) The effect of aphidicolin-synchronization (*n* = 4) from DPI-1 to DPI-6 on the percentage and absolute number yield of GFP^high^ iCMs. * *p* < 0.05; ** *p* < 0.01, *** *p* < 0.001 vs. control.

Our time-lapse recordings showed that iCMs at the initiation stage (the arrow-pointed cell in frame i of Figure 1A) expressed a low amount of αMHC-GFP (GFP^low^) and gradually turned into brighter GFP^+^ cells (GFP^high^) along with the progress of reprogramming (18 h later in frame ii to iV of Figure 1A). Our FACS data also disclosed that iCMs have varying intensities of GFP fluorescence (Figure 3A), suggesting that the intensity of GFP fluorescence might indicate different stages of reprogramming achieved in individual iCMs. We then gated all reprogrammed-αMHC-GFP^+^ cells at DPI-2, which were newly reprogrammed in theory, as a GFP^low^ sub-population (Figure 3A) and gated remaining αMHC-GFP^+^ cells with more intense GFP-fluorescence as a GFP^high^ sub-population. We found that GFP^high^ iCMs contained 13.3 ± 1.6% cells that were stained positively for cardiac troponin T, which is significantly more than that in GFP^low^ iCMs (6.6 ± 1.1%, *n* = 5, *p* = 0.0005, Figure 3B). Importantly, a significantly smaller portion of EdU^+^ cells in GFP^high^ iCM-population than that in GFP^low^ population at DPI-7 (*n* = 3) and DPI-10 (*n* = 6) (Figure 3C), suggesting that a bigger portion of GFP^high^ iCMs had exited cell cycle. We then sorted out GFP^low^ and GFP^high^ populations and found that, compared to GFP^low^ cells, GFP^high^ iCMs expressed many cardiac genes at a significantly higher level, including *Atp2a2*, *Myl7*, *Actc1*, and *Ryr2* (*n* = 6, Figure 3D and Figure S3A,B), while the expression of *Mki67*, a proliferation marker gene, was significantly lower in GFP^high^ cells. These results demonstrated that a more advanced degree of reprogramming had been achieved in GFP^high^ iCMs. Importantly, S-phase synchronization (*n* = 6), but no other cell-cycle phase synchronizations (*n* = 3), at DPI-1 significantly increased the portion of GFP^high^ iCMs at DPI-7 (Figure 3E).

We next investigated how S-phase synchronization influences the yield of iCMs along the process of GMT-reprogramming and found that, unlike at DPI-1, S-phase synchronization from DPI-2 to DPI-6 had no inhibition on the yield of total αMHC-GFP^+^ iCMs (*n* = 3, Figure 3F and Figure S3C,D). Importantly, S-phase synchronization from DPI-2 to DPI-5 actually yielded two to four times more number of GFP^high^-iCMs than the unsynchronized control (*n* = 4, Figure 3G and Figure S3E), suggesting that S-phase synchronization accelerated the early progression of GMT-reprogramming.

**Figure 4:**
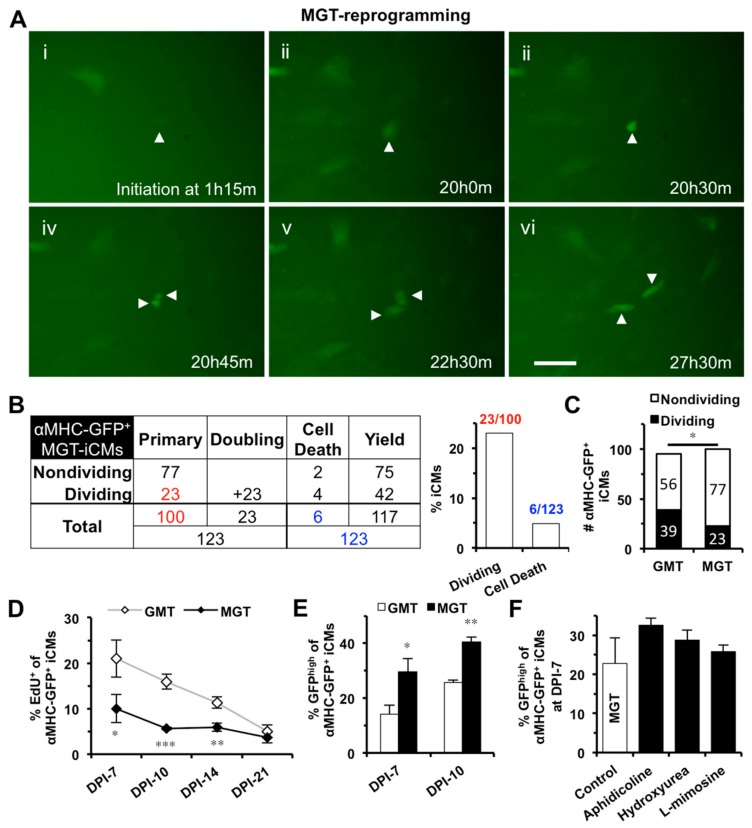
S-phase synchronization could not further improve the enhanced reprogramming of polycistronic construct (MGT). (**A**) Representative images of time-lapse recording showing that one MGT-iCM (arrowhead) divided into two daughter iCMs. Scale bar indicates 50 μm. (**B**) A table summarizing the time-lapse result of all three batches of MGT-reprogrammed αMHC-GFP^+^ iCMs. Numbers in red were used to calculate the percentage of dividing primary MGT-iCMs and numbers in blue for the percentage of cell death among total iCMs, which were summarized in the bar graph. (**C**) Time-lapse recordings revealed significantly less dividing cells among MGT-iCMs than GMT-iCMs. (**D**) EdU assays showed that MGT-iCMs exited cell cycle earlier than GMT-iCMs (*n* = 4). (**E**) MGT-reprogramming yielded more GFP^high^ iCMs than GMT-reprogramming at DPI-7 (*n* = 7) and DPI-10 (*n* = 3). (**F**) S-phase synchronization at DPI-1 had no significant improvement on MGT-reprogramming (*n* = 4). * *p* < 0.05, ** *p* < 0.01, *** *p* < 0.001 vs. control.

We next investigated the effect of S-phase synchronization on iCM-reprograming mediated by a polycistronic construct (MGT), which expresses an optimal stoichiometry of three reprogramming factors and could yield a better efficiency and a better quality of iCM-reprogramming in mouse cardiac fibroblasts than GMT monocistronic constructs [10]. We found that GMT- and MGT-reprogramming of MEFs yielded a similar number of iCMs at DPI-3 through DPI-10 (*n* = 3, Figure S4A). Our 48-h time-lapse recordings also captured cell division and cell death in MGT-reprogrammed iCMs (MGT-iCMs) from DPI-2 to DPI-4 (Figure 4A,B, Movie S2); however, the number of dividing cells was significantly less in MGT-iCMs than in GMT-iCMs (Figure 4C). Consistently, there were significantly less EdU^+^ cells in MGT-iCMs than in GMT-iCMs within the first two weeks of reprogramming (*n* = 4, Figure 4D). Moreover, MGT-reprogramming was processed faster and yielded a significantly higher portion of GFP^high^ iCMs than GMT-reprogramming at DPI-7 (*n* = 7) and DPI-10 (*n* = 3) (Figure 4E). These results demonstrated that an advanced progression with enhanced cell-cycle exit has been achieved in iCMs reprogrammed by polycistronic MGT. Importantly, we found that S-phase synchronization failed to further increase the percentage of GFP^high^ population among MGT-iCMs (*n* = 4, Figure 4F), suggesting that the facilitated progression of GMT-reprogramming by S-phase synchronization was mediated through a mechanism of enhanced cell-cycle exit.

## 3. Discussion

In this study, we focused on understanding the early progression of iCM-reprogramming and found that iCMs did go through cell division at the early stage of reprogramming and ultimately exited cell cycle during the process of reprogramming. Importantly, we found that post-infection S-phase synchronization facilitated the early progression of GMT-reprogramming and yielded more GFP^high^ iCMs through a mechanism of enhanced cell-cycle exit.

Cell cycle includes two critical phases—a synthesis phase (S-phase) of accurate DNA duplication and a mitosis phase of chromosome segregation—that are preceded by two gap phases, G1- and G2-phase respectively. The epigenetic status at S-phase suppresses global RNA transcription and protein synthesis, with the exception of histone proteins [21], however, we observed that the activation of αMHC-GFP could be also initiated at S-phase, suggesting that iCM-reprogramming is conducted throughout different phases of cell cycle and might continue through more than one cell cycle. Indeed, our time-lapse recordings revealed that iCM-reprogramming was processed and continued through at least one cell-cycle as shown by cell division of iCMs following αMHC-GFP activation in both monocistronic GMT- and polycistronic MGT-mediated reprogramming. Consistently, a recent study of single-cell transcriptomics reconstructed a path of cell-fate conversion from fibroblast to iCMs and disclosed a population of early-stage reprogrammed iCMs that underwent cell division [28]. Therefore, iCMs remain active in cell cycle at the early stage of reprogramming.

Moreover, our study also demonstrated that iCMs exited cell cycle at a later stage of reprogramming and S-phase synchronization following the initiation of reprogramming could enhance cell-cycle exit in GMT-iCMs. Interestingly, the enhanced cell-cycle exit by S-phase synchronization was accompanied with an improved progression of GMT-reprogramming and yielded significantly more GFP^high^ iCMs, which achieved a more advanced reprogramming than GFP^low^ cells. This might be due to that cell-cycle exit prevents a dilution of GMT expression in dividing iCMs and subsequently induces high cardiac gene expression and better reprogramming. This facilitated progression is also validated in iCM-reprogramming of polycistronic MGT [10], which accelerated cell-cycle exit and yielded more GFP^high^ iCMs. Because of this accelerated progression of MGT-reprogramming, S-phase synchronization failed to further increase the GFP^high^ portion in MGT-iCMs, indicating that a common mechanism of enhanced cell-cycle exit is shared by both methods. Consistently, the active cell-cycle status at the later stages of reprogramming was found to negatively correlate to the maturity of reprogrammed iCMs [20,28] and iCM-reprogramming was significantly suppressed in an immortalized cardiac fibroblast line, which never exits cell cycle [28]. These all together demonstrate that cell-cycle exit is an essential process of iCM-reprogramming. In addition, our time-lapse recordings also found that some iCMs reprogrammed by either GMT or MGT underwent cell death, possibly apoptosis, which could explain why inhibitors of ROCK signaling increased the yield of reprogrammed iCMs in a previous study [16]. 

One limitation is that our study focuses on cell-cycle regulation during the early progression of iCM-reprogramming. It is unknown how much the overall functional maturation of iCMs could be achieved at later stages of reprogramming by the strategy of accelerated cell-cycle exit. The initiation of iCM-reprogramming at late G1- and S-phase and the enhancement of S-phase synchronization, found in this study, suggest that the epigenetics of different cell-cycle phases might play a critical role to initiate iCM-reprogramming and S-phase epigenetics might benefit iCM reprogramming, although more comprehensive study is needed to validate it in the future. In addition, further study is also required to show a benefit of S-phase synchronization on iCM reprogramming in adult cardiac fibroblasts. 

## 4. Materials and Methods

### 4.1. Animal Use Protocol

All animal protocols have been reviewed and approved by Case Western Reserve University Institutional Animal Care and Use Committee (Approval#: 2015-0058; Approval Date: 22 April 2015).

### 4.2. Mouse Embryonic Fibroblast Isolation

Mouse embryonic fibroblasts (MEFs) were isolated from transgenic αMHC-GFP mouse embryos (E12.5–13.5) with modifications in a previously reported method [17]. Briefly, embryos were extracted from pregnant mice under sterile conditions and only embryos with αMHC-GFP^+^ expression in the hearts were used for MEF isolation. To prevent any cardiomyocyte contamination, embryonic hearts were carefully removed as well as other internal organs and head. Embryos were chopped into small pieces (1–2 mm^3^) and incubated in 2 mL of 0.125% trypsin/EDTA per embryo for 20 min in a water bath at 37 °C. Every 5 min, tissue pieces were pipetted up and down 5–10 times to dissociate the tissue. Then, 1 mL additional Trypsin per embryo was added and incubated for approximately 10 min until there is no visible tissue chunks. To stop enzyme digestion, an equal volume of dulbecco’s modified eagle medium (DMEM) with 10% fetal bovine serum (FBS) (Hyclone, Logan, UT, USA; Fisher Scientific, Pittsburgh, PA, USA) was added and cells were filtered through a 40 µM cell strainer (Corning Falcon, Tewksbury, MA, USA; Fisher Scientific) followed by centrifugation at 1500 rpm for 3 min. The pellet was dissolved in MEF medium (DMEM with 10% heat-inactivated FBS) and cultured in a 10 cm dish per three embryos for 2–3 days until they reach nearly 100% confluency. Primary MEFs were passaged freshly for reprogramming or stored in liquid nitrogen for later use.

### 4.3. Direct Cardiac Reprogramming and Flow Cytometry

For iCM-reprogramming, retroviruses were generated as previously reported [2,10]. Briefly, pMX retroviral Gata4, Mef2c, or Tbx5 plasmid [2] or polycistronic Mef2c-P2A-Gata4-T2A-Tbx5 (MGT) plasmid [10] was transfected into PlatE cells (at ~90% confluence) with FugeneHD transfection reagent (Promega, Madison, WI, USA) as per manufacturer’s protocol. The next day, media was refreshed with PlatE media (DMEM with 10% FBS). Viruses were harvested 48 h after transfection and filtered through 0.45 µM low protein-binding filter (Nalgene, New York, NY, USA; Thermo Fisher, Waltham, MA, USA). MEFs, which were seeded into 6-well plates ~24 h in advance at the density of 120,000 cells/well without any gelatin coating, were infected with a mixture of three viruses of Gata4, Mef2c, and Tbx5 (GMT, 0.5 mL each) or 0.5 mL MGT for 24 h in the presence of polybrene (8 µg/mL, Millipore, Cleveland, OH, USA). Infected MEFs were maintained in cardiac reprogramming media, which is consisted of DMEM/M199 (4:1) with 10% heat-inactivated FBS, NEAA (Gibco, Gaithersburg, MD, USA), and l-glutamine (Gibco), with media changing every 2 to 3 days. For evaluating reprogramming efficiency at either day 7 post-infection (DPI-7) or DPI-10, iCMs were harvested by 0.05% trypsin/EDTA and dissolved in FACS buffer (2 mM EDTA, 5% FBS in phosphate-buffered saline (PBS). The percentage and absolute number of αMHC-GFP^+^ iCMs reprogrammed by monocistronic GMT (GMT-iCMs) or polycistronic MGT (MGT-iCMs) were evaluated by BD Accuri C6 flow cytometer (BD Biosciences, San Jose, CA, USA).

### 4.4. Cell-Cycle Synchronization

For cell-cycle synchronization, GMT-retrovirus-infected MEFs were incubated with thymidine (2 mmol/L, Sigma, Washington, DC, USA), lovastatin (25 µmol/L, Sigma), nocodazole (50 ng/mL, Sigma), aphidicolin (2µg/mL, Sigma), hydroxyurea (2 mmol/L, Sigma), l-mimosine (0.5 mmol/L, Sigma), or serum-free DMEM media at DPI-1 for 24 h. After synchronization, MEFs were washed with PBS for three times to remove drugs and were cultured in cardiac reprogramming media for iCM-reprogramming.

### 4.5. Time-Lapse Imaging of iCM-Reprogramming

To understand the early progression of iCM-reprogramming, retrovirus-infected MEFs were cultured in a micro-incubator (STXG-WSKMX, Tokai Hit, Shizuoka, Japan) at 37 °C, 5% CO_2_ and were monitored from DPI-2 to DPI-4 by DMi8 Leica fluorescent microscope (Leica Microsystems, Wetzlar, Germany). Brightfield and GFP-fluorescent images were recorded from the same sites every 15 min for 48 h. A three-second-exposure time was purposely set up for GFP-fluorescence recording so that the initiation of iCM-reprogramming with very faint GFP-fluorescence could be recognized. Recorded pictures were analyzed by MetaMorph software (Molecular Devices, San Jose, CA, USA) to assess cell division in reprogrammed-iCMs and non-reprogrammed MEFs.

### 4.6. Cell-Cycle Assays

For analysis of cell-cycle phases, plain MEFs with or without cell-cycle synchronization were incubated with EdU (10 µmol/L) for 2 h and then harvested for staining with anti-EdU antibodies (1:200) and propidium iodide (0.08 µg/µL, Sigma) using Click-iT™ Plus EdU Alexa Fluor™ 647 Flow Cytometry Assay Kit (Fisher Scientific, Pittsburgh, PA, USA) with some modifications in the protocol. Briefly, the cells were harvested in 0.05% trypsin/EDTA, washed with PBS once, and fixed by 4% paraformaldehyde (PFA) in pellet, followed by staining with EdU at room temperature and propidium iodide at 37 °C, respectively. The cells were kept on ice in propidium iodide staining solution prior to cell-cycle analysis by BD Accuri C6 flow cytometer.

For the analysis of cell division in iCMs, GMT-retrovirus-infected MEFs were incubated with EdU (10 µmol/L) for 24 h and harvested for immunostaining with anti-EdU (1:200) and anti-GFP-Fluorescein isothiocyanate antibodies (1:100, Novus Biologicals, Littleton, CO, USA). EdU^+^/αMHC-GFP^+^ GMT-iCMs were analyzed by BD Accuri C6 (BD Biosciences, San Jose, CA, USA).

### 4.7. Flow Cytometry of Cardiac Troponin-T^+^ iCMs

Reprogrammed iCMs were harvested at DPI-7 and fixed with 4% PFA. For immunostaining of αMHC-GFP and cardiac troponin-T (cTnT), cells were incubated with conjugated antibodies of rabbit anti-GFP-FITC (1:400, Invitrogen, Carlsbad, CA, USA) and mouse anti-cTnT (1:400, Thermo Scientific, Waltham, MA, USA) at 4 °C overnight, and then incubated with fluorescence conjugated secondary antibodies (Invitrogen). Cells were analyzed by BD Accuri C6.

### 4.8. Western Blot Analysis

To estimate the expression level of reprogramming factors, total proteins were extracted from MEFs at various time points after GMT-retrovirus infection and used for a standard western blot assay with antibodies of Gata4 (1:5000, Santa Cruz Biotechnology, Santa Cruz, CA, USA), Mef2c (1:5000, Aviva Systems Biology, San Diego, CA, USA), and Tbx5-Flag (1:500, Thermo Scientific). β-Actin (1:1000, Sigma) or Gapdh (1:1000, Santa Cruz Biotechnology) were used as the housekeeping gene control. Pierce ECL Plus Chemiluminescence Detection Kit (Thermo Scientific) was used to detect the proteins.

### 4.9. Real-Time qPCR Assay

Reprogrammed GFP^low^ and GFP^high^ iCMs (~10,000 cells) were sorted out separately by HAPS1 cell sorter (iCyt, Sony Biotechnology, San Jose, CA, USA) and used for reverse transcription to generate cDNA by CellsDirect One-Step qRT-PCR Kit (Invitrogen). After pre-amplification with pooled primers, standard quantitative PCR assays were performed by a 7300 Real-Time PCR system (Applied Biosystems, Foster City, CA, USA). The expression levels of cardiac and proliferation genes (Table S2) were normalized to a housekeeping gene *Gapdh*.

### 4.10. Statistical Analyses

All data were analyzed with at least three biological replicates and expressed as mean ± SEM. Statistical significance was examined by one-way analysis of variance (ANOVA) and subsequent student’s *t*-test (two-way paired or unpaired) or chi-square test, accordingly. *p* values of <0.05 were recognized as statistically significant. * *p* < 0.05, ** *p* < 0.01, *** *p* < 0.001.

## 5. Conclusions

In summary, our study provides direct evidence that iCMs actually go through cell division at an early reprogramming stage and exit cell cycle along the process of reprogramming. Cell-cycle exit is one critical event or an indicator of the transition into a more advanced reprogramming. Enhanced cell-cycle exit by S-phase synchronization promotes the early progression of iCM-reprogramming and induces an advanced reprogrammed degree in iCMs earlier. Our study improves the understanding of iCM-reprogramming process by enlightening potential roles of cell-cycle regulation during iCM-reprogramming, which will guide us to further optimize this nascent reprogramming approach for future translational applications.

## Supplementary Materials

Supplementary materials can be found at http://www.mdpi.com/1422-0067/19/5/1364/s1.

## Abbreviations

iCM	Induced cardiomyocytes
MEF	Mouse embryonic fibroblasts
αMHC	α-myosin heavy chain
GMT	Gata4, Mef2c, and Tbx5 (monocistronic constructs)
GMT-iCMs	αMHC-GFP^+^ iCMs reprogrammed by GMT
MGT	Mef2c-P2A-Gata4-T2A-Tbx5 (polycistronic construct)
MGT-iCMs	αMHC-GFP^+^ iCMs reprogrammed by MGT
DPI	Days post-infection
EdU	5-ethynyl-20-deoxyuridine
