# Supplementary material for: S-phase Synchronization Facilitates the Early Progression of Induced-Cardiomyocyte Reprogramming through Enhanced Cell-Cycle Exit
Source: Int J Mol Sci. 2018 May 4;19(5):1364. doi: 10.3390/ijms19051364 (PMC5983785; doi:10.3390/ijms19051364)
Supplement: Supplementary file 1 [file ijms-19-01364-s001.zip › Supplementary Documents.pdf]

SUPPLEMENTARY FIGURES

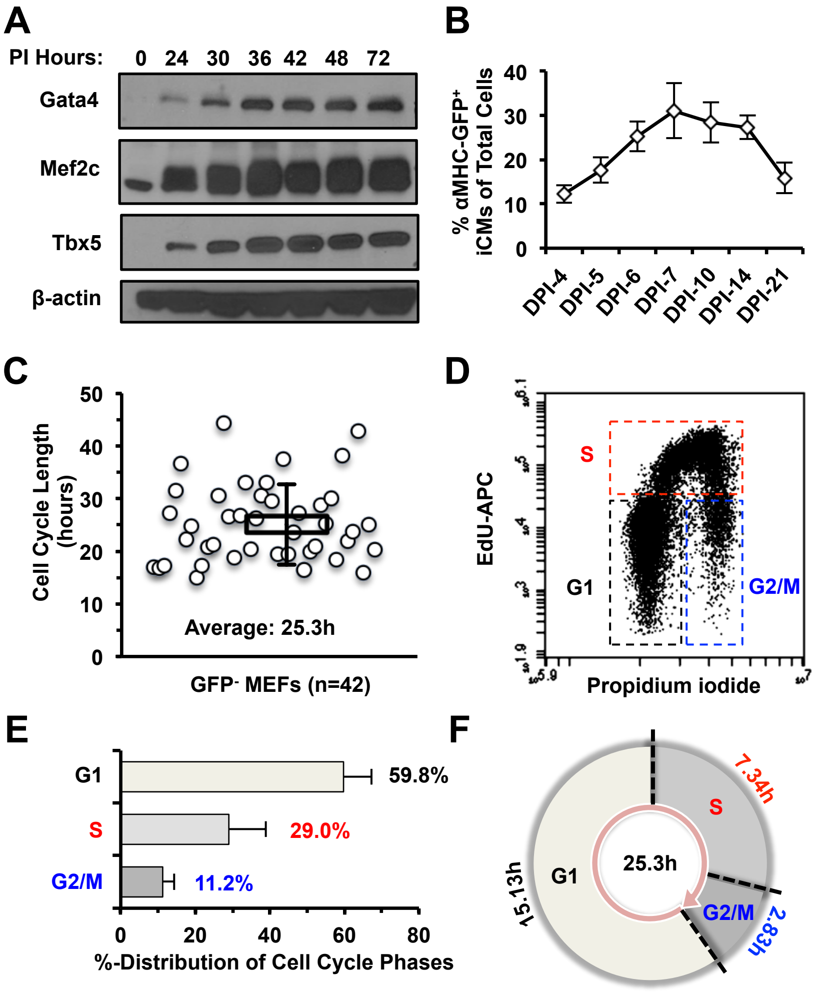

Figure S1. **iCM Reprogramming by monocistronic Gata4, Mef2c, and Tbx5 (GMT) and cell cycle length of MEFs**. **A)** Representative western blot image shows the expression of Gata4, Mef2c, and Tbx5 in MEFs at different post-infection (PI) hours. **B)** The percentage of αMHC-GFP+ GMT-iCMs from DPI-4 to DPI-21 (n=3). **C)** Non-reprogrammed MEFs, which had two consecutive cell divisions in the time-lapse recordings (n=42), had an average of 25.3±7.4 hours cell-cycle length. **D)** Representative FACS plot of EdU assay with two-hour EdU-labeling showing a distribution of cell-cycle phases in MEFs. **E)** The average percentages of G1-, S-, and G2/M-phase in MEFs (n=4). **F)** MEFs had an average of 15.2-hour G1 phase, 7.3-hour S phase, and 2.8-hour G2/M phase.

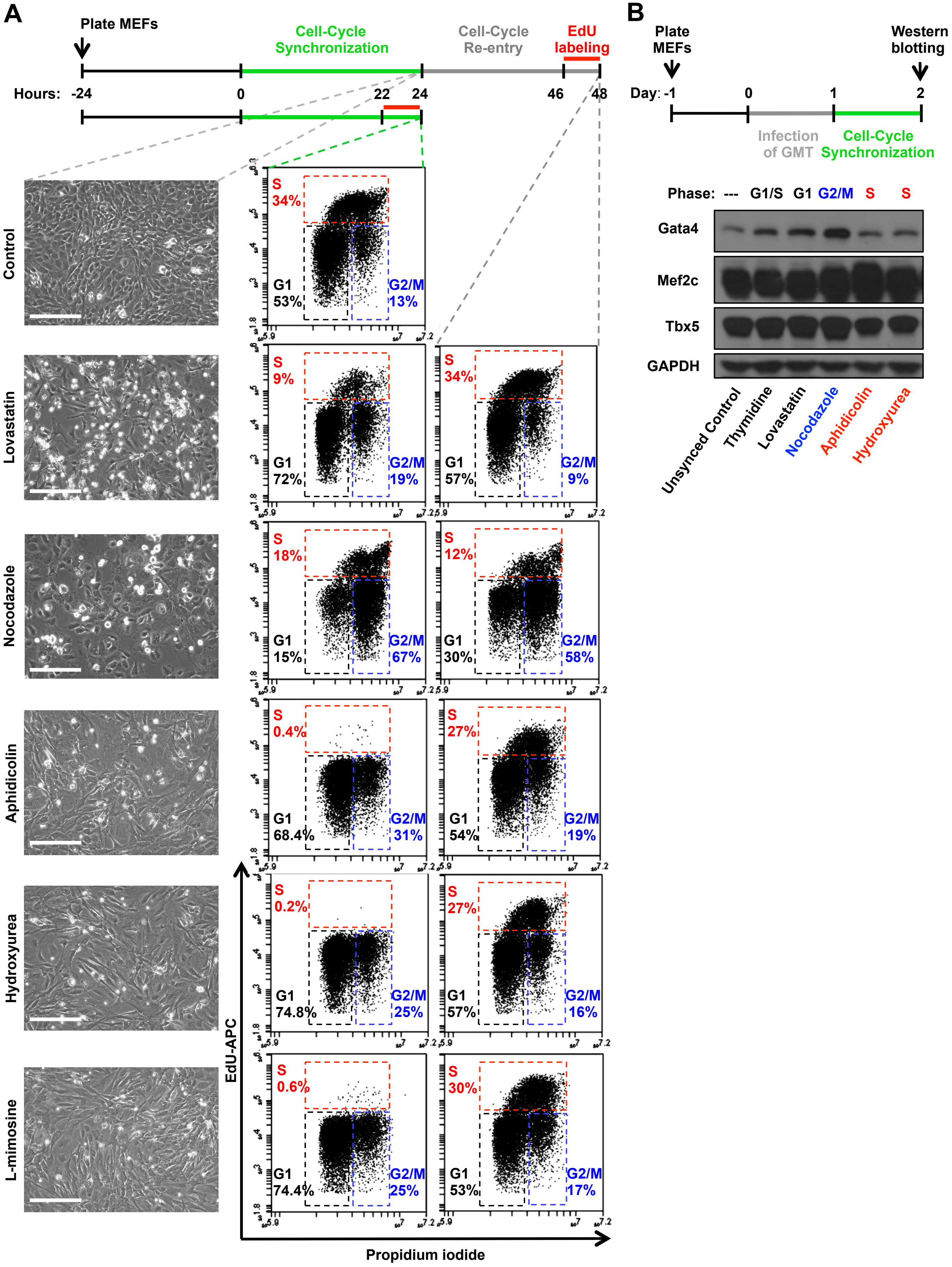

Figure S2. Cell-cycle synchronization and reentrance of MEFs. A) Representative pictures and FACS plots show that un-reprogrammed MEFs were synchronized into different cell-cycle phases by relevant treatments. Synchronized MEFs reentered cell cycle 24 hours after releasing from synchronization (Right). Scale bars indicate 50μm. **B**) Protein expressions of Gata4, Mef2c, and Tbx5 in MEFs were not inhibited by any treatments of cell-cycle synchronization.

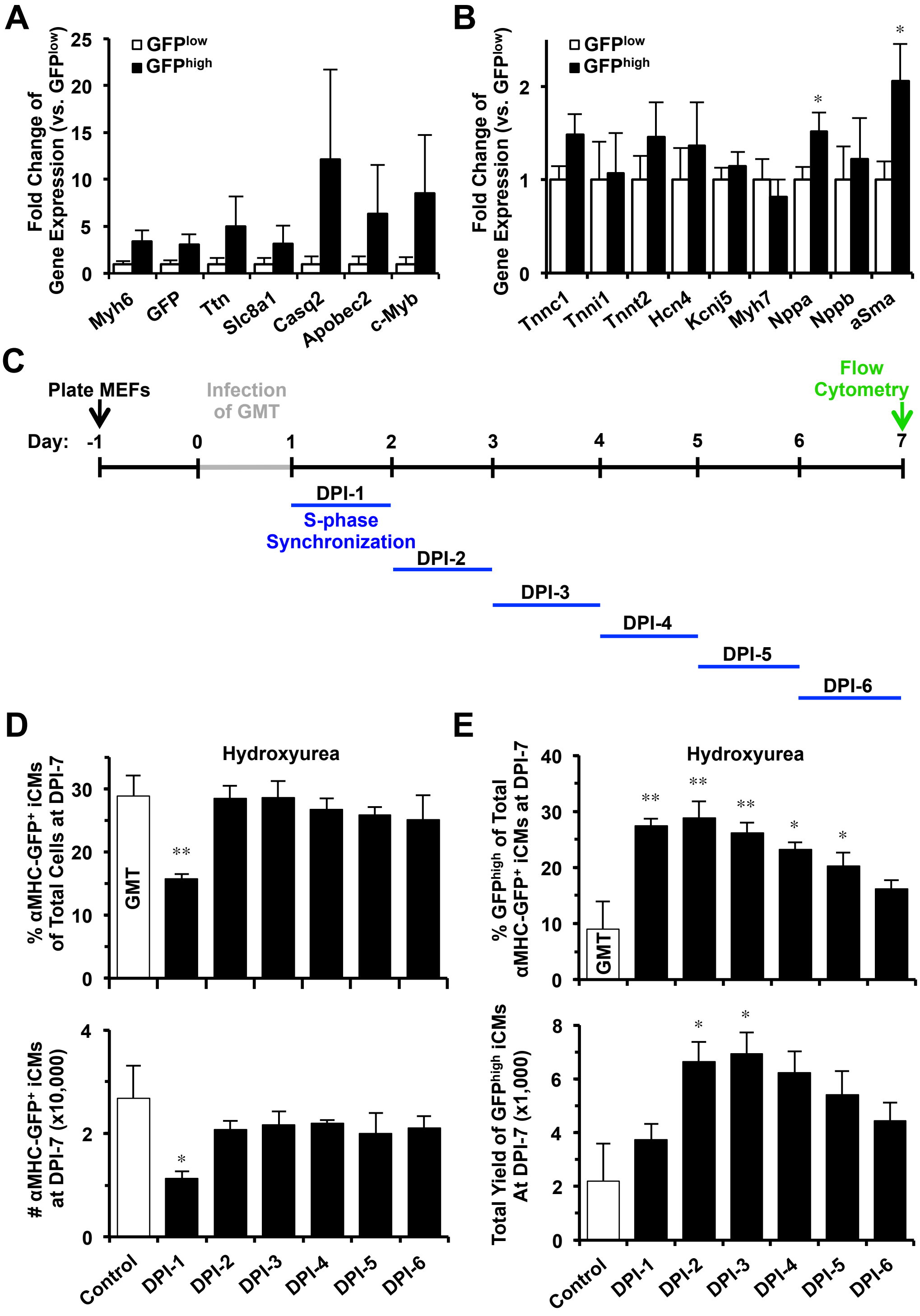

Figure S3. S-phase synchronization increases the yield of GFP^{high} iCMs. A-B) Comparisons of cardiac gene expressions between GFP^{low} and GFP^{high} iCMs (n=6). *p<0.05 vs. GFP^{low}. **C**) Experimental design of S-phase synchronization from day-1 post-infection (DPI-1) to DPI-7. **D)** The effect of S-phase synchronization by hydroxyurea (n=3) from DPI-1 to DPI-6 on the percentage and absolute number of αMHC-GFP^{+} GMT-iCMs. **E**) The effect of hydroxyurea-synchronization (n=4) from DPI-1 to DPI-6 on the percentage and total yield of GFP^{high} iCMs. *p<0.05; **p<0.01, ***p<0.001 vs. control.

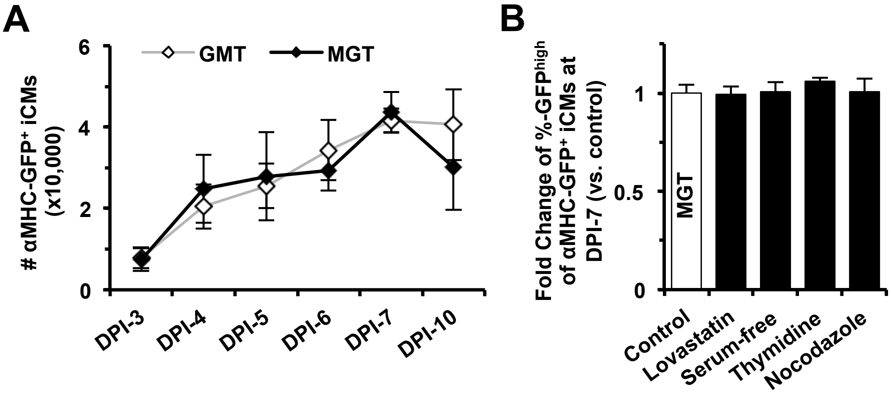

Figure S4. The influence of cell-cycle synchronization on polycistronic MGT-reprogramming. A) Polycistronic MGT successfully reprogrammed MEFs and yielded a similar number of αMHC-GFP^{+} iCMs as monocistronic GMT (n=3). **B)** Cell-cycle synchronizations of G1 (lovastatin and serum-free), G1/S (thymidine), and G2/M (nocodazole) at DPI-1 had no significant influence on the yield of GFP^{high} MGT-iCMs

SUPPLEMENTARY TABLES

Table S1. Time from cell division back to reprogramming initiation in GMT-iCMs

Total # of time-lapsed αMHC-GFP+ iCMs	Dividing iCMs used for analysis	Time from cell division back to reprogramming initiation (hours)
Batch-1 (64 iCMs)	#1	4.25
	#2	19
	#3	20
	#4	12.5
	#5	11.25
	#6	14.5
	#7	13.75
	#8	13.5
	#9	16.5
	#10	9.25
	#11	2
	#12	15
	#13	18
	#14	5.5
	#15	12.25
	#16	4.5
	#17	5.5
	#18	6.25
Batch-2 (26 iCMs)	#19	14.75
	#20	16.5
	#21	14.5
	#22	10
	#23	14.75
	#24	4.5
Batch-3 (44 iCMs)	#25	2.25
	#26	18.25
	#27	19.25
	#28	21.5
	#29	7.75
	#30	18.75
	#31	4.75
	#32	5.75
	#33	10.75
	#34	14

Table S2. qRT-PCR primers for gene expression analysis of iCMs

Gene	Primer sets		Product size (bp)
Atp2a2	F<br>R	5'- TCTACGTGGAACCTTTGCCG -3'<br>5'- GCTGCACACACTCTTTACCG -3'	162
Myl7	F<br>R	5'- GGTCCCATCAACTTCACCGT -3'<br>5'- AAGGCACTCAGGATGGCTTC -3'	86
Actc1	F<br>R	5'- TGCCATGTATGTCGCCATCC -3'<br>5'- CACCATCGCCAGAATCCAGA -3'	86
Ryr2	F<br>R	5'- ACGGCGACCATCCACAAAG -3'<br>5'- AAAGTCTGTTGCCAAATCCTTCT -3'	67
Myh6	F<br>R	5'- GCCCAGTACCTCCGAAAGTC -3'<br>5'- GCCTTAACATACTCCTCCTTGTC -3'	110
GFP	F<br>R	5'- GGACGACGGCAACTACAAGA -3'<br>5'- AAGTCGATGCCCTTCAGCTC -3'	87
Ttn	F<br>R	5'- CCGATGTTTACGCAGCCGTTA -3'<br>5'- TCAAAGGTTGCGGTACTACCC -3'	62
Slc8a1	F<br>R	5'- CTTCCCTGTTTGTGCTCCTGT -3'<br>5'- AGAAGCCCTTTATGTGGCAGTA -3'	78
Casq2	F<br>R	5'- GCCCAACGTCATCCCTAACA -3'<br>5'- CCCATTCAAGTCGTCTTCCCA -3'	133
Apobec2	F<br>R	5'- GATCTTCCGCCCTTCGAGATT -3'<br>5'- TCTGTACTTCGACCACATAGCA -3'	130
c-Myb	F<br>R	5'- AGACCCCGACACAGCATCTA -3'<br>5'- CAGCAGCCCATCGTAGTCAT -3'	81
Tnnc-1	F<br>R	5'- GGAGCTGTCGGATCTCTTCC -3'<br>5'- GGCCATCGTTGTTCTTGTCAC -3'	155
Tnni-1	F<br>R	5'- ACCATGCCGGAAGTTGAGAG -3'<br>5'- GAATGCGCTCCGAGAGGTAA -3'	151
Tnnt-2	F<br>R	5'- ACAGAGGAGGCCAACGTAGA -3'<br>5'- AAGTTGGGCATGAAGAGCCT -3'	113
Hcn4	F<br>R	5'- ACTCCTGGGGGAAGCAGTAT -3'<br>5'- GCCGATGAACATGGCATAGC -3'	158
Kcnj5	F<br>R	5'- ATACTCCTTCTGGTGCAGGC -3'<br>5'- GCTCTCTTCTTTGGCTGGCT -3'	95
Myh7	F<br>R	5'- ACTGTCAACACTAAGAGGGTCA -3'<br>5'- TTGGATGATTTGATCTTCCAGGG -3'	114
Nppa	F<br>R	5'- CCCTCGGAGCCTACGAAGAT -3'<br>5'- TGTTGCAGCCTAGTCCACTC -3'	80
Nppb	F<br>R	5'- GATCCGTCAGTCGTTTGGGC -3'<br>5'- AAAGAGACCCAGGCAGAGTCA -3'	98
MKi67	F<br>R	5'- ATCATTGACCGCTCCTTTAGGT -3'<br>5'- GCTCGCCTTGATGGTTCCT -3'	104
aSMA	F<br>R	5'- ATCACCAACTGGGACGACAT -3'<br>5'- CATACATGGCTGGGACATTG -3'	175
Gapdh	F<br>R	5'- AGGTCGGTGTGAACGGATTTG -3'<br>5'- TGTAGACCATGTAGTTGAGGTCA -3'	123

SUPPLEMENTARY MOVIE LEGENDS

Movie S1. A time-lapse recording movie of GFP-fluorescence images (Left) and overlay of GFP and brightfield images (Right) showing that GMT-iCMs underwent cell division from DPI-2 to DPI-4.

Movie S2. A time-lapse recording movie of GFP-fluorescence images showing that MGT-iCMs underwent cell division from DPI-2 to DPI-4.
